# Comparative study on the effects of crystalline L-methionine and methionine hydroxy analogue calcium supplementations in the diet of juvenile Pacific white shrimp (*Litopenaeus vannamei*)

**DOI:** 10.3389/fphys.2023.1067354

**Published:** 2023-01-30

**Authors:** Lu Zheng, Yuechong Liu, Yanmei Zhang, Bingying Xu, Gladstone Sagada, Zhixuan Wang, Cong Chen, Xiandong Lang, Jiaonan Zhang, Qingjun Shao

**Affiliations:** ^1^ Aqua-feed and Nutrition Laboratory, College of Animal Sciences, Zhejiang University, Hangzhou, China; ^2^ Shandong NHU Amino Acid Co., Ltd, Weifang, China; ^3^ Fujian Province Key Laboratory of Special Aquatic Formula Feed, Fujian Tianma Science and Technology Co., Ltd, Fuqing, China

**Keywords:** growth performance, anti-oxidative capacity, protein synthesis, immunity, methionine, *Litopenaeus vannamei*

## Abstract

An 8-week feeding trial was conducted to evaluate the effects of L-methionine and methionine hydroxy analogue calcium (MHA-Ca) supplements in low-fishmeal diet on growth performance, hepatopancreas morphology, protein metabolism, anti-oxidative capacity, and immunity of Pacific white shrimp (*Litopena* *eus vannamei*). Four isonitrogenous and isoenergetic diets were designed: PC (203.3 g/kg fishmeal), NC (100 g/kg fishmeal), MET (100 g/kg fishmeal +3 g/kg L-methionine) and MHA-Ca (100 g/kg fishmeal +3 g/kg MHA-Ca). White shrimp (initial body weight 0.23 ± 0.00 g, 50 shrimp per tank) were allocated to 12 tanks and divided among 4 treatments in triplicates. In response to L-methionine and MHA-Ca supplementations, the shrimp exhibited higher weight gain rate (WGR), specific growth rate (SGR), condition factor (CF), and lower hepatosomatic index (HSI) compared to those fed the NC diet (*p* < 0.05). The WGR and SGR of shrimp fed L-methionine and MHA-Ca showed no difference with those in the PC diet (*p* > 0.05). Both of L-methionine and MHA-Ca supplementary diets significantly decreased the malondialdehyde (MDA) levels of shrimp when compared with the NC diet (*p* < 0.05). L-methionine supplementation improved the lysozyme (LZM) activity and total antioxidant capacity (T-AOC) of shrimp, while the MHA-Ca addition elevated the reduced glutathione (GSH) levels in comparison with those fed the NC diet (*p* < 0.05). Hypertrophied blister cells in hepatocytes were observed in shrimp fed the NC diet, and alleviated with L-methionine and MHA-Ca supplementations. Shrimp fed the MET and MHA-Ca diets had higher mRNA expression levels of target of rapamycin (*tor*) than those fed the NC diet (*p* < 0.05). Compared to the NC group, dietary MHA-Ca supplementation upregulated the expression level of cysteine dioxygenase (*cdo*) (*p* < 0.05), while L-methionine supplementation had no significant impact (*p* > 0.05). The expression levels of superoxide dismutase (*sod*) and glutathione peroxidase (*gpx*) were significantly upregulated by L-methionine supplemented diet in comparison with those in the NC group (*p* < 0.05). Overall, the addition of both L-methionine and MHA-Ca elevated the growth performance, facilitated protein synthesis, and ameliorated hepatopancreatic damage induced by plant-protein enriched diet in *L. vannamei*. L-methionine and MHA-Ca supplements enhanced anti-oxidants differently.

## Introduction

Pacific white shrimp (*Litopenaeus vannamei*) is preferred by several consumers and aquaculture farmers because of its nutritional value, tenderness, palatability, and high tolerance to salinity and temperature (Zhou et al., 2013; Gao et al., 2017). Fishmeal is an optimal protein source in aquafeeds because of its high level of protein, balanced amino acid composition and taste (Liu et al., 2021). The sustainable development of *L. vannamei* farming faces challenges due to escalating fishmeal cost and limited amounts of wild fish resources. Thus, reducing the reliance on fishmeal is increasingly inevitable, and plant protein sources are promising alternatives to fishmeal in intensive aquaculture in the view of sustainability, as advocated by the Food and Agriculture Organization of the United Nations (FAO, 2018). Derived from soy, soy protein concentrate has a more reasonable amino acid composition and palatability, as well as fewer anti-nutritional factors, despite the lack of sulfur-containing amino acids (Cruz-Suárez et al., 2009; Bauer et al., 2012; Lu et al., 2021). Among the sulfur-containing amino acids, methionine is considered as the first limiting essential amino acid (EAA) with biological functions in protein synthesis, transmethylation reactions, synthesis of cysteine and cystine, as well as anti-oxidative ability (Mastrototaro et al., 2016; Lu et al., 2021; Zhou et al., 2021). Studies have demonstrated that methionine deficiency may lead to oxidative stress, liver damage and growth retardation in animals (Bin et al., 2017; Lee et al., 2019). Chen et al. (2018) reported that methionine deficiency led to lower weight gain rate, protein retention efficiency and higher feed conversion rate of *L. vannamei* when 25% fishmeal was replaced with soybean protein concentrate. As such, to cover the methionine requirements of animals, the exploration of methionine supplementation under low-fishmeal aquafeeds is being extensively researched.

Methionine products have been mainly supplemented in the following forms: crystalline DL-methionine, crystalline L-methionine and DL-2-hydroxy-4-methylthiobutanoic acid (methionine hydroxy analogue, MHA). The MHA comes in the form of hydroxy analogue free acid (MHA-FA) or methionine hydroxy analogue salts such as methionine hydroxy analogue calcium (MHA-Ca) (Huyghebaert, 1993; Nunes et al., 2014; Powell et al., 2017; Niu et al., 2018). As products of chemical synthesis, both crystalline DL-methionine and MHA are racemic mixture of two enantiomers (D-isomers and L-isomers) (Sveier et al., 2001). Crystalline amino acids are characterized by a high leaching rate in the aquatic environment, specifically for slow-feeding crustaceans, and have a lower utilization rate due to the rapid absorption and metabolism of free amino acids by animals in comparison with amino acids obtained from digested protein (Chi et al., 2011; Gu et al., 2013). As an organic acid salt rather than an amino acid, MHA exhibits lower water solubility when compared to crystalline methionine, although it cannot be utilized by animals until it is converted into L-methionine after a series of metabolic transformations. In addition, there are distinguished differences between crystalline methionine and MHA in absorption, transport and metabolism *in vivo* as the result of their different chemical properties (Maenz and Engele-Schaan, 1996; Pham Thi Ha To et al., 2020; To et al., 2021). Recent research has shown that the transport of L-methionine relies on multiple apical sodium-dependent/independent high/low-affinity transporters, while the transport of MHA is mediated by proton-dependent monocarboxylate transporters of the SLC16 family and apical sodium monocarboxylate transporters of the SLC5 family (To et al., 2021). The application effects of crystalline methionine and analogue products are still controversial in aquaculture. Some studies have shown that these methionine products are similar in their ability to satisfy the qualitative requirements of aquatic species without adverse effects (Goff and Gatlin, 2004; Forster and Dominy, 2006; Zhou et al., 2018), while others have indicated that analogue products supplementation was not comparable with crystalline methionine (Keembiyehetty and Gatlin, 1995; Zhao et al., 2017; Chen et al., 2018).

Currently, supplementation of methionine products in low-fishmeal diet of *L. vannamei* has been a hot research topic. To the best of our knowledge, the enhanced growth performance and antioxidant enzyme response with methionine products supplementation in low-fishmeal diet of *L. vannamei* have been demonstrated (Gu et al., 2013; Niu et al., 2018; Xie et al., 2018; Wang et al., 2019), yet the physiological mechanism underlying these characteristics is poorly understood. Likewise, the assessment of these different methionine products on the immunity is a field of great interest. Machado et al. (2015) presented that L-methionine supplementation in the diet of European seabass (*Dicentrarchus labrax*) improved immune response to inflammation induced with UV-inactivated *Photobacterium damselae* subsp. *Piscicida* (*Phdp*). The addition of methionine in low-fishmeal aquafeeds represents a strategy to boost immune ability and welfare, as well as provide a lower-cost alternative to replace antibiotics.

With the progress of production technology, the properties of artificial methionine products have been improved, and the purity of MHA-Ca in product has been elevated from 95.0% to 98.8%. Hence, it is important to continuously assess the efficacy of different methionine products *in vivo*. The objectives of this study were to compare the impacts of L-methionine and MHA-Ca products in low-fishmeal diets on the growth performance, anti-oxidative ability, methionine metabolism, protein synthesis, and immunity of *L. vannamei*.

## Materials and methods

### Diets design and preparation

Based on nutritional requirements of *L. vannamei*, four isonitrogenous (40.21% crude protein) and isoenergetic (17.89 kJ/g gross energy) diets were designed and formulated as shown in Table 1. The dietary design including the supplemental amount of methionine products in this work referred to previous study in our laboratory (Wang et al., 2019). The positive control (PC) diet was formulated with fishmeal at 203.3 g/kg while the negative control (NC) diet contained a lower proportion of fishmeal (100 g/kg) attained with higher soy protein concentrate. At the same fishmeal level as the NC diet, 3 g/kg crystalline L-methionine and 3 g/kg MHA-Ca with 87.8% MHA (purity of 98.8%) were supplemented in the other two diets and designated as MET and MHA-Ca diets, respectively. Among the four diets, fishmeal, soybean meal, fermented soybean meal, soybean protein concentrate and shrimp meal were the main protein sources. Aspartic acid: glycine (1:1) and α-starch were added to realize isonitrogenous and isoenergetic requirements in the four diets. The proximate composition of these ingredients is presented in supplementary material (Supplementary Table S1). The AA profiles and MHA-Ca content of the four diets are shown in Table 2. All ingredients were ground adequately through a 178 μm mesh before being weighed and mixed thoroughly. A feed machine (Modle HKJ-218; HUARUI) was used to transform the homogenized mixture into pellets with a particle size of 1.2 mm. Subsequently, all pellets were steamed for 10 min and dried for 72 h under an air conditioner. All diets were stored at −20 °C until used.

**TABLE 1:** Formulation and proximate composition of the experimental diets (g/kg diet).

Ingredients	Diets			
	PC	NC	MET	MHA-Ca
Fishmeal^a^	203.3	100.0	100.0	100.0
Soybean meal^a^	100.0	100.0	100.0	100.0
Fermented soybean meal^a^	100.0	100.0	100.0	100.0
Soybean protein concentrate^a^	70.0	170.0	170.0	170.0
Shrimp meal^a^	50.0	50.0	50.0	50.0
Squid liver meal^a^	30.0	30.0	30.0	30.0
Chicken meal^a^	60.0	60.0	60.0	60.0
Wheat flour	220.0	220.0	220.0	220.0
Beer yeast	40.0	40.0	40.0	40.0
Fish oil^a^	20.8	30.0	30.0	30.0
Maize oil	0.3	0.0	0.0	0.0
Soybean lecithin	20.0	20.0	20.0	20.0
L-carnitine	2.0	2.0	2.0	2.0
*Yarrowia lipolytica*	2.0	2.0	2.0	2.0
α-Starch	19.6	0.0	0.0	0.0
L-Methionine^b^	0.0	0.0	3.0	0.0
MHA-Ca^c^	0.0	0.0	0.0	3.0
Carboxymethyl cellulose	5.0	5.0	5.0	5.0
Carrageenan	3.7	3.7	3.7	3.7
Ascorbic phosphate ester	1.0	1.0	1.0	1.0
Vitamin premix^d^	3.0	3.0	3.0	3.0
Mineral premix^e^	5.0	5.0	5.0	5.0
L-Lysine	1.8	3.0	3.0	3.0
Asp: Gly (1:1)	0.0	3.0	0.0	0.0
Ca(H_2_PO_4_)_2_	20.5	22.0	22.0	22.0
Zeolite powder	21.0	21.0	21.0	21.0
Butyrin	1.0	1.0	1.0	1.0
α-Cellulose	0.0	8.3	8.3	8.3
Proximate composition				
Moisture (%)	8.18	8.04	8.02	8.04
Crude protein (%)	43.28	43.79	43.26	42.91
Crude lipid (%)	6.80	6.79	6.98	6.85
Crude ash (%)	11.83	10.79	10.68	10.73
Gross energy (kJ/g)^f^	18.04	18.28	18.31	18.25

^a^ Purchased from Jin Jia Co., Ltd, Hangzhou, China.

^b^ Purchased from Sangon Biotech Co., Ltd, Shanghai, China.

^c^ Provided by Shandong NHU, Amino Acid Co., Ltd, China.

^d^ Composition of vitamin premix (mg/kg): α-tocopherol, 80; retinyl acetate, 40; cholecalciferol, 0.1; menadione, 15; niacin, 165; riboflavin, 22; pyridoxine HCl, 40; thiamin mononitrate, 45; D-Ca pantothenate, 102; folic acid, 10; vitamin B_12_, 0.9; inositol, 450; ascorbic acid, 150; Na menadione bisulphate, 15; thiamin, 5; choline chloride, 320 and p-aminobenzoic acid, 50.

^e^ Composition of mineral premix (mg/kg): Na_2_SiO_3_, 0.4; CaCO_3_, 544.9; NaH_2_PO_4_·H_2_O, 200; KH_2_PO_4_, 200; MgSO_4_·7H_2_O, 10; MnSO_4_·H_2_O, 4; CuCl_2_·2H_2_O, 2; ZnSO_4_·7H_2_O, 12; FeSO_4_·7H_2_O, 12; NaCl, 12; KI, 0.1; CoCl_2_·6H_2_O, 0.1; Na_2_MoO_4_·2H_2_O, 0.5; AlCl_3_·6H_2_O, 1; and KF, 1.

^f^ Gross energy was calculated by using the factors 23.6 kJ/g for protein, 39.5 kJ/g for lipid, and 17.2 kJ/g for carbohydrate. Carbohydrate was calculated as 100–(protein + lipid + ash + moisture).

**TABLE 2:** The amino acid composition and MHA-Ca content of experimental diets (g/kg dry matter).

	Diets			
	PC	NC	MET	MHA-Ca
EAA				
Threonine	18.00	17.97	17.56	18.06
Valine	20.99	21.12	21.00	21.45
Methionine	8.82	7.77	9.86	7.96
Isoleucine	15.06	15.83	15.38	16.15
Leucine	35.00	36.18	35.82	36.47
Phenylalanine	17.57	18.06	18.07	17.01
Lysine	26.42	25.94	25.77	26.47
Histidine	9.13	9.69	9.52	9.82
Arginine	21.50	22.88	22.37	23.12
NEAA				
Alanine	26.58	25.76	25.01	25.34
Aspartate	46.23	47.80	48.52	49.32
Cysteine	5.73	5.62	5.88	5.79
Glutamic acid	72.06	72.16	72.67	74.42
Glycine	18.76	17.42	17.54	18.12
Proline	2.55	2.53	2.49	2.60
Serine	18.52	18.60	18.96	19.85
Tyrosine	3.88	3.82	3.89	3.79
Other methionine source				
MHA-Ca	−	−	−	2.11

EAA, essential amino acids; NEAA, Non-essential amino acids.

### Experimental procedure

Healthy *L. vannamei* were obtained from Zhejiang Mariculture Research Institute (Wenzhou, China). The feeding trial was conducted in Xixuan Fishery Science and Technology Island (Zhoushan, China). Before the feeding experiment, shrimp were fed commercial diet for 4 weeks to acclimate to the culture conditions. Following a 24-h fasting, 600 healthy shrimp (initial body weight: 0.23 ± 0.00 g per shrimp) were randomly selected and transferred into 12 500-L fibreglass tanks (50 shrimp per tank), and each diet was assigned to triplicate tanks. During the 8-week feeding trial, shrimp were reared in a flow-through system, supplied with filtered seawater continuously and fed to apparent satiation three times a day (7:00, 13:00 and 18:00) under natural illumination. Any uneaten feed and feces were removed every day. The water conditions were as follows: temperature, 26–28°C; pH, 8.0–8.3; dissolved oxygen, ≥6.5 mg/L; salinity, 27.2–30.0 g/L; and total ammonia nitrogen, 0.3–0.5 mg/L. These rearing conditions are well within the acceptable levels of *L.vannamei*.

### Sample collection

After the 8-week feeding trial and the 24-h fasting period, all surviving shrimp were weighed and counted. 15 shrimp from each tank were selected randomly for the measurement of whole length, body weight and hepatopancreas weight, and then 10 shrimp from each tank were collected for proximate chemical composition analysis. Hemolymph was extracted from the pericardial sinus located at the base of the first abdominal segment using a 1-mL disposable sterile syringe and blended with an equal volume of pre-cooled anticoagulant (0.34 M sodium chloride, 0.16 M disodium hydrogen phosphate, 0.04 M sodium dihydrogen phosphate, 0.02 M EDTA, pH 7.4). Plasma for biochemical analysis was collected after centrifugation at 3500 *g*, 4°C for 10 min and frozen in liquid nitrogen immediately. Hepatopancreas for subsequent biochemical and genetic analysis were frozen in liquid nitrogen after dissection. Then, samples collected in the liquid nitrogen were stored at − 80°C. For histological observation, hepatopancreas were taken and fixed in 10% (v/v) formaldehyde solution for 24 h, then transferred to 70% ethanol solution until further use.

### Analytical methods for proximate composition

According to the standards of Association of Official Analytical Chemists (Kavanagh, 2010), moisture, crude protein, crude lipid and ash contents of the four diets and whole shrimp were determined. Following the description in previous research (Yuan et al., 2011; Chen et al., 2018; Niu et al., 2018), the amino acid composition of diets and whole shrimp were determined by an automatic amino acid analyzer (Hitachi LA8080, Tokyo, Japan) after acid hydrolysis in HCl solution, while methionine measurement was conducted by performic acid oxidation methods. The MHA-Ca measurement followed the protocol in Balschukat et al. (1988).

### Biochemical assays

Hepatopancreas were homogenized with PBS (1:9) and centrifuged immediately at 4000 × *g*, 4°C for 10 min. The obtained supernatants were used to measure biochemical parameters with relevant kits. Specifically, total protein was measured with BCA Protein Assay Kit (A045-3, Jiancheng Bioengineering Institute, Nanjing, China). Total antioxidant capacity (T-AOC), catalase (CAT), superoxide dismutase (SOD), reduced glutathione (GSH), and malondialdehyde (MDA) in supernatants were analyzed with A015–1, A007-1-1, A001–1, A006-1-1, and A003-1 kits (Jiancheng Bioengineering Institute, Nanjing, China), respectively. In addition, total cholesterol (T-CHO), lysozyme (LZM), aspartate aminotransferase (AST), and alanine aminotransferase (ALT) in plasma were detected using A111-1-1, A050-1-1, C010-1-1, and C009-1-1 kits (Jiancheng Bioengineering Institute, Nanjing, China), respectively. The phenoloxidase (PO) determination was as referenced by Lin et al. (2012) with kits (G0146W, Suzhou Grace Biotechnology Co., Ltd., China).

### H&E staining of hepatopancreas

Prefixed hepatopancreas were put in a series of graded ethanol for dehydration, treated with xylene, and embedded in paraffin wax (Gao et al., 2019). Paraffin blocks containing samples were cut into 5-μm-thick sections, stained with hematoxylin and eosin (H&E), and viewed under an optical microscope (Nikon Ni-U, Japan).

### RNA extraction and quantitative real-time PCR analysis (qRT-PCR)

Total RNA of hepatopancreas was extracted with Trizol reagent (Vazyme Biotech Co., Ltd., Nanjing, China) as previously described (Liu et al., 2018). The concentration and quality of RNA were examined with NanoDrop 2000 spectrophotometer (Thermo Fisher Scientific, Delaware, USA) and 1% agarose gel electrophoresis. Only RNA sample with OD260/280 ratio between 1.8 and 2.0, and with OD260/230 ratio ≥2.0 were selected to synthesize cDNA using reverse transcription with a Prime Script™ RT Reagent Kit with gDNA Eraser (Takara, China). The specific primers for qRT-PCR were designed by premier primer 5.0 software, and the nucleotide sequences were referenced from NCBI (https://www.ncbi.nlm.nih.gov/) and previous studies. The stability of all primers were examined using 1% agarose gel electrophoresis, and the verification of primers specificity followed protocols in Chang et al. (2018); Bustin et al. (2010). A detailed description of primers can be found in Table 3, with *β-actin* as the internal control. The cDNA was diluted with DEPC-treated water (1:5), and mixed with primer and reagents TB Green^®^ Premix Ex Taq™ (Takara, China) for qRT-PCR, which was conducted in the LightCycler 480 apparatus (Roche, Switzerland). The reaction profile for qRT-PCR was set up as follows: initial denaturation at 95°C for 1 min, 45 cycles of 5 s denaturation, 15 s annealing at 60°C and 20 s extension at 72°C, following a melting curve and cooling to 37°C. All RT-qPCR reactions were performed in triplicates and the relative expression levels of selected genes were calculated by 2^−ΔΔCT^ (Livak and Schmittgen, 2001).

**TABLE 3:** Sequences of primers used in *L. vannamei*.

Target genes	Forward primer sequence (5′–3′)	Reverse primer sequence (5′–3′)	Product size (bp)	Source
*mat2a*	AGG​CAC​CCA​AGA​AGG​CAT​CCC​A	GCT​GCT​CCT​TTC​CGC​TCC​ACT​G	144	XM_027377879.1
*cdo*	AAG​TAG​CCA​CCT​TGG​ACG​ACC​T	TAG​AGG​TAT​GCG​AAG​GTC​CCC​A	228	KP325604
*tor*	TGCCAACGGGTGGTAGA	GGGTGTTTGTGGACGGA	181	XM_027372359
*rag c*	GGA​TGA​TGA​CCA​CTA​CCG​CTC​G	GAA​CAG​TGT​CTC​ATT​GGG​GGT​C	220	XM_027357730
*4ebp1*	ATG​TCT​GCT​TCG​CCC​GTC​GCT​CGC​C	GGT​TCT​TGG​GTG​GGC​TCT​T	226	XM_027367939.1
*sod*	GCA​ATG​AAT​GCC​CTT​CTA​CC	CAG​AGC​CTT​TCA​CTC​CAA​CG	199	Cardona et al. (2015)
*cat*	TAC​TGC​AAG​TTC​CAT​TAC​AAG​ACG	GTA​ATT​CTT​TGG​ATT​GCG​GTC​A	285	Cardona et al. (2015)
*gpx*	GGC​ACC​AGG​AGA​ACA​CTA​C	CGA​CTT​TGC​CGA​ACA​TAA​C	102	Cardona et al. (2015)
*lzm*	TGTTCCGATCTGATGTCC	GCTGTTGTAAGCCACCC	121	AY170126
*casp 3*	AGT​TAG​TAC​AAA​CAG​ATT​GGA​GCG	TTG​TGG​ACA​GAC​AGT​ATG​AGG​C	156	KC660103.1
*β-actin*	GAG​CAA​CAC​GGA​GTT​CGT​TGT	CAT​CAC​CAA​CTG​GGA​CGA​CAT​GGA	68	AF300705

*mat2a*, methionine adenosyltransferase II, alpha; *cdo*, cysteine dioxygenase; *tor*, target of rapamycin; *rag c*, Ras-related GTP-binding protein C; *4ebp1*, eukaryotic translation initiation factor 4 E binding protein 1; *sod*, superoxide dismutase; *cat*, catalase; *gpx*, glutathione peroxidase; *lzm*, lysozyme; *casp 3*, caspase 3.

### Statistical analysis

The growth performance and feed utilization parameters were calculated as follows:

Weight gain rate (WGR, %) = (final body weight–initial body weight)/initial body weight × 100.

Specific growth rate (SGR, %/day) = (ln final body weight–ln initial body weight)/day × 100.

Survival rate (SR, %) = final shrimp number/initial shrimp number × 100.

Condition factor (CF, g/cm^3^) = body weight/body length^3^ × 100

Hepatosomatic index (HSI, %) = hepatopancreas weight/whole body weight × 100

Feed conversion rate (FCR) = dry feed intake/(final body weight − initial body weight)

All data were presented as means ± standard deviation (SD) and analyzed for normality and homogeneity in SPSS 19.0 software. The differences among treatments were determined by one-way analysis of variance (ANOVA), followed by Duncan’s multiple-range test at *p* < 0.05.

## Results

### Growth performance and feed utilization of *L. vannamei*

The growth and feed utilization parameters of shrimp in response to the four different diets are presented in Table 4. Co**m**pared to the NC group, shrimp fed the PC diet exhibited higher WGR, SGR and CF as well as lower HSI (*p* < 0.05). The supplementation of L-methionine and MHA-Ca (MET and MHA-Ca diets) led to higher WGR, SGR and CF, as well as lower HSI in comparison with those fed the NC diet (*p* < 0.05), while the HSI of shrimp fed the MHA-Ca diet was higher than those fed the PC and MET diets (*p* < 0.05). No difference of WGR and SGR was observed among shrimp fed the MET, MHA-Ca and PC diets (*p* > 0.05).There was no distinction in CF and HSI between the PC and MET groups (*p* > 0.05). Furthermore, shrimp from the MHA-Ca group showed the highest CF (*p* < 0.05). The SR and FCR of shrimp did not differ significantly among the four groups (*p* > 0.05).

**TABLE 4:** Growth performance and feed utilization of *L. vannamei* fed the experimental diets.

Indexes	Diets			
	PC	NC	MET	MHA-Ca
WGR (%)	3167.32 ± 122.02^b^	2603.23 ± 264.02^a^	3079.97 ± 169.35^b^	3308.30 ± 69.84^b^
SGR (%/day)	6.39 ± 0.10^b^	6.10 ± 0.18^a^	6.39 ± 0.11^b^	6.48 ± 0.10^b^
SR (%)	98.67 ± 2.31	97.33 ± 3.06	96.67 ± 4.16	98.00 ± 2.83
CF (g/cm^3^)	0.76 ± 0.01^b^	0.72 ± 0.00^a^	0.80 ± 0.02^b^	0.85 ± 0.04^c^
HSI (%)	4.38 ± 0.49^a^	5.86 ± 0.46^c^	4.40 ± 0.55^a^	4.90 ± 0.70^b^
FCR	1.35 ± 0.08	1.29 ± 0.01	1.38 ± 0.05	1.29 ± 0.00

WGR, weight gain rate; SGR, specific growth rate; SR, survival rate; CF, condition factor; HSI, hepatosomatic index; FCR, feed conversion rate. Values are presented as mean ± SD (*n* = 3), values with unlike superscript letters are significantly different (*p* < 0.05).

### Whole-body composition and amino acid profiles of *L. vannamei*

The effects of the four different diets on the whole-body composition and amino acid profiles of *L. vannamei* are shown in Table 5 and Table 6, respectively. No significant difference was found in moisture, crude protein, crude lipid and ash contents of shrimp among the four groups (*p* > 0.05). Shrimp fed the PC diet presented the highest methionine content among all groups, and those fed the MET and MHA-Ca diets showed significantly higher levels of methionine content than the NC group (*p* < 0.05). The supplementation of MHA-Ca increased the valine content of shrimp significantly (*p* < 0.05), with some notable increases in those fed the PC and MET diets as well. The contents of threonine, isoleucine, leucine, phenylalanine, lysine histidine, arginine, and non-essential amino acids (NEAA) of shrimp in different groups were not significantly affected (*p* > 0.05)

**TABLE 5:** Whole-body composition of *L. vannamei* fed the experimental diets (% dry matter).

Indexes	Diets			
	PC	NC	MET	MHA-Ca
Moisture	76.84 ± 0.96	76.69 ± 0.90	76.74 ± 0.31	76.17 ± 0.44
Crude protein	74.69 ± 1.03	72.35 ± 2.02	71.09 ± 2.26	72.58 ± 2.02
Crude lipid	5.69 ± 1.15	4.38 ± 0.69	4.90 ± 1.44	3.85 ± 1.77
Crude ash	13.65 ± 0.76	13.60 ± 0.89	13.36 ± 0.50	13.57 ± 0.51

Values are presented as mean ± SD (n = 3), values with unlike superscript letters are significantly different (*p* < 0.05).

**TABLE 6:** The amino acid composition in the whole body of *L. vannamei* fed the experimental diets (g/kg dry matter).

	Diets			
	PC	NC	MET	MHA-Ca
EAA				
Threonine	27.84 ± 0.04	28.62 ± 0.38	27.72 ± 0.59	28.52 ± 0.44
Valine	32.26 ± 0.85^ab^	31.71 ± 0.40^a^	32.20 ± 0.46^ab^	33.15 ± 0.46^b^
Methionine	14.50 ± 0.14^c^	13.75 ± 0.21^a^	14.07 ± 0.12^b^	14.27 ± 0.06^bc^
Isoleucine	24.78 ± 0.43	24.50 ± 0.37	24.16 ± 0.74	25.40 ± 1.15
Leucine	51.54 ± 1.21	52.10 ± 0.72	51.56 ± 0.63	52.92 ± 0.97
Phenylalanine	28.22 ± 0.53	28.20 ± 0.09	27.88 ± 0.52	28.66 ± 0.56
Lysine	47.31 ± 1.01	48.30 ± 1.16	46.71 ± 1.36	48.59 ± 1.15
Histidine	10.47 ± 0.50	11.03 ± 0.37	10.99 ± 0.45	11.75 ± 0.70
Arginine	45.44 ± 0.66	45.57 ± 0.20	45.76 ± 0.13	46.91 ± 0.75
NEAA				
Alanine	49.94 ± 0.85	49.96 ± 2.15	50.21 ± 1.12	51.66 ± 1.45
Aspartate	77.04 ± 2.00	78.96 ± 1.14	77.08 ± 1.15	77.80 ± 2.66
Cysteine	2.13 ± 0.06	2.16 ± 0.02	2.16 ± 0.02	2.11 ± 0.05
Glutamic acid	100.77 ± 1.05	102.04 ± 3.13	101.67 ± 2.50	104.03 ± 2.38
Glycine	50.39 ± 1.48	50.13 ± 0.88	51.31 ± 1.19	53.75 ± 4.25
Proline	23.27 ± 0.22	23.24 ± 0.27	23.16 ± 0.32	23.26 ± 0.50
Serine	26.72 ± 0.51	25.41 ± 0.65	25.92 ± 0.60	26.94 ± 0.70
Tyrosine	15.10 ± 0.59	14.85 ± 0.37	14.99 ± 0.12	15.12 ± 0.51

Values are presented as mean ± SD (*n* = 3). Values with unlike superscript letters are significantly different (*p* < 0.05).

### Biochemical parameters of *L. vannamei*

The biochemical parameters in plasma and hepatopancreas of *L. vannamei* after feeding with the four diets are displayed in Table 7. In the plasma, the highest LZM activity was observed in shrimp fed the MET diet, followed by shrimp fed the PC diet (*p* < 0.05). No significant difference in T-CHO levels was observed among the four groups (*p* > 0.05). Shrimp fed the NC diet had the highest AST activity, albeit significantly higher than only the PC group (*p* < 0.05). No significant difference in ALT of *L. vannamei* occurred among the four groups (*p* > 0.05). In the hepatopancreas, the T-AOC level of shrimp fed the MET diet was significantly higher than that of the NC group (*p* < 0.05). Shrimp from the MHA-Ca group had significantly higher GSH when compared with the NC group (*p* < 0.05). The highest MDA was observed in shrimp fed the NC diet, and dietary L-methionine and MHA-Ca supplementation reduced the MDA levels significantly (*p* < 0.05). The results indicated no difference in SOD activity among all groups (*p* > 0.05).

**TABLE 7:** Biochemical parameters of juvenile *L. vannamei* fed the experimental diets.

Parameters	Diets			
	PC	NC	MET	MHA-Ca
Plasma				
T-CHO (mmol/L)	2.13 ± 0.19	1.57 ± 0.33	2.18 ± 0.68	1.53 ± 0.28
LZM (U/ml)	50.57 ± 7.96^b^	32.18 ± 3.98^a^	66.67 ± 10.53^c^	45.98 ± 3.98^ab^
AST (U/l)	5.35 ± 2.28^a^	9.20 ± 0.41^b^	8.74 ± 0.00^ab^	7.72 ± 1.17^ab^
ALT (U/l)	9.14 ± 1.84	9.80 ± 2.06	10.02± 2.00	8.46 ± 0.58
Hepatopancreas				
T-AOC (U/mg prot)	2.58 ± 1.42^ab^	1.47 ± 0.35^a^	6.57 ± 1.04^b^	1.92 ± 0.75^a^
CAT (U/mg prot)	3.06 ± 0.49^b^	1.19 ± 0.09^a^	1.80 ± 0.44^a^	1.88 ± 0.37^a^
SOD (U/mg prot)	2.66 ± 1.75	4.20 ± 1.48	2.64 ± 0.07	3.68 ± 2.93
GSH (mg/g prot)	16.83 ± 2.82^b^	3.72 ± 0.11^a^	7.54 ± 2.74^a^	15.72 ± 0.93^b^
MDA (nmol/mg prot)	1.74 ± 0.58^a^	9.56 ± 1.42^b^	1.65 ± 0.27^a^	1.58 ± 0.11^a^

T-CHO, total cholesterol; LZM, lysozyme; AST, aspartate aminotransferase; ALT, alanine aminotransferase; T-AOC, total antioxidant capacity; CAT, catalase; SOD, superoxide dismutase; GSH, reduced glutathione; MDA, malondialdehyde. Values are presented as mean ± SD (n = 3), values with unlike superscript letters are significantly different (*p* < 0.05).

### Histological morphology of hepatopancreas

**FIGURE 1:**
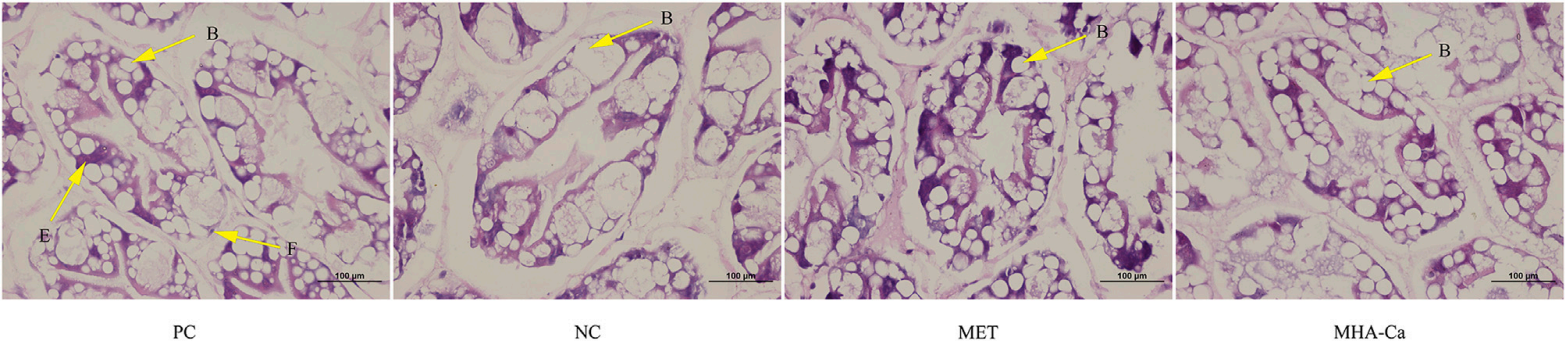
Representative histological photomicrographs of hepatopancreas from *L. vannamei* fed different diets (H&E staining, magnification × 400). The letters B, E, F in tubules represent blister cells (B cells), embryonic cells (E cells) and fibrillar cells (F cells), respectively.

Histological sections of hepatopancreas tubules from *L. vannamei* fed the four diets are presented in Figure 1. The blister cells (B-cell), embryonic cells (E cells) and fibrillar cells (F cells) in the hepatocytes were identified in shrimp fed the four diets. Shrimp fed the NC diet had hypertrophied B-cell in hepatopancreas, while L-methionine and MHA-Ca supplementations alleviated the hypertrophy. *L. vannamei* fed the PC, MET and MHA-Ca diets exhibited normal hepatocyte structures with regular arrangement and no abnormality in tubules.

### Gene expression in hepatopancreas

**FIGURE 2:**
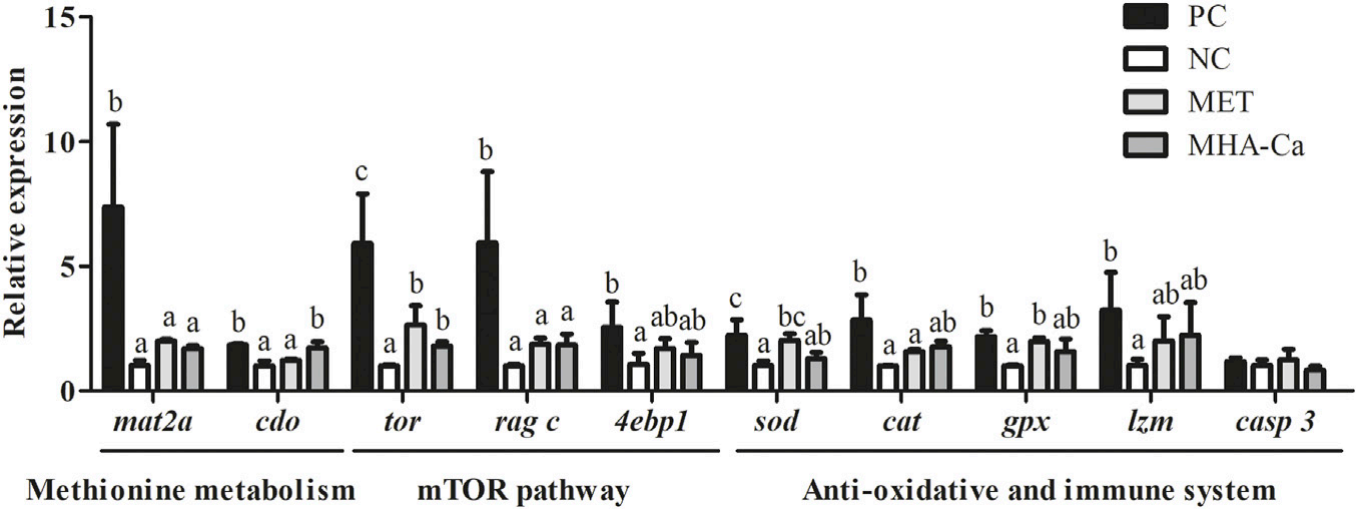
Relative expression of methionine metabolism, mTOR signaling pathway, anti-oxidative and immune system related genes in hepatopancreas of *L. vannamei* fed with four diets. All gene expression of the NC group was standardized as about 1. All expression values are normalized with *β-actin* (n=3), with SD denoted by vertical bars. Different letters over columns indicate significantly different (*p* < 0.05).

The expression of genes involved in methionine metabolism, mTOR signaling pathway, anti-oxidative and immune system in hepatopancreas are presented in Figure 2. Shrimp fed the PC diet showed significantly higher expression levels of methionine adenosyltransferase II, alpha (*mat2a*) and cysteine dioxygenase (*cdo*) in comparison with those fed the NC diet (*p* < 0.05). The addition of L-methionine had no significant implication on the expression levels of *mat2a* and *cdo* compared to the NC group (*p* > 0.05). With the supplementation of MHA-Ca, the expression level of *cdo* was upregulated significantly (*p* < 0.05). In the mTOR signaling pathway, compared with shrimp fed the NC diet, those fed the PC diet exhibited significantly higher expression levels of target of rapamycin (*tor*) and Ras-related GTP-binding protein C (*rag c*) significantly in the mTOR signaling pathway (*p* < 0.05). Both L-methionine and MHA-Ca supplements significantly upregulated the expression of *tor* (*p* < 0.05). The expression of *rag c* was not influenced by L-methionine or MHA-Ca (*p* > 0.05). The expression level of eukaryotic translation initiation factor 4 E binding protein 1 (*4ebp1*) in the MET and MHA-Ca groups showed no differences with another two groups (*p* > 0.05). In the anti-oxidant system, the expression levels of *sod*, *cat* and glutathione peroxidase (*gpx*) in shrimp fed the PC diet were markedly higher than those in the NC group (*p* < 0.05). A significant upregulation of *sod* and *gpx* was observed in the MET group (*p* < 0.05). Comparatively, the addition of MHA-Ca had no significant effect on *sod* and *gpx* expression (*p* > 0.05). The expression of *cat* in the MET and MHA-Ca groups showed no significant difference compared to the NC group (*p* > 0.05). The expression levels of *lzm* in shrimp fed the MET and MHA-Ca diets increased slightly, albeit without statistical significance. No significant difference in *casp 3* was observed among four groups (*p* > 0.05).

## Discussion

In order to reduce the reliance on fishmeal, the feasibility of balancing amino acid composition with synthetic amino acids supplementation in plant-protein diets has been well researched in aquaculture (Hidalgo et al., 1987; Fauconneau, 1988; Ren et al., 2019; Li et al., 2021). Several studies have demonstrated that L-methionine and MHA-Ca can be used as methionine supplements and improve the growth performance of animals (Keembiyehetty and Gatlin, 1995; Goff and Gatlin, 2004; Luo et al., 2005; Powell, et al., 2017), which was also demonstrated in this study. After the 8-week feeding trial, dietary methionine supplementation had a positive effect on the growth performance of *L. vannamei*, by improving the WGR, SGR and CF, regardless of the source of methionine. Niu et al. (2018) reported that 0.3% DL-methionine (8.2 g/kg methionine content in diet) improved WGR, SGR and SR of *L. vannamei* significantly. Furthermore, the results of Xie et al. (2018) also indicated that dietary 0.3% DL-methionine supplementation (10.1 g/kg methionine content in diet) could notably improve WGR and SGR of *L. vannamei* when compared with shrimp fed a low-methionine diet (7.3 g/kg methionine content in diet). The comparison of L-methionine and MHA-Ca was conducted in Goff and Gatlin, (2004). They revealed that both L-methionine and MHA-Ca performed with equivalent efficiency on elevating the growth performance of red drum (*Sciaenops ocellatus*). In contrast, MHA-Ca showed less efficiency in supporting the growth of channel catfish when compared to L-methionine (Robinson et al., 1978). In this study, L-methionine and MHA-Ca were added in equal amounts rather than as equal amounts of methionine as in most previous studies, and the results indicated similar growth-promoting effects of both on *L. vannamei*. When comparing the effects of the two additives on growth performance, it is important to consider their characteristics such as solubility, digestion sites and pathways, and purity, as well as the aquatic animal species.

Hepatopancreas, analogous to liver and pancreas in mammals, plays a key role in nutrient metabolism and health for crustaceans (Verri et al., 2001). Our results found higher HSI levels in shrimp fed the NC diet because of methionine deficiency. High HSI is one of the consequences of liver swelling (Li et al., 2016). Gu et al. (2013) demonstrated that the deprivation of methionine in plant-protein diets caused liver swelling from fat accumulation or disorder in sulphur metabolism. The current study detected a decrease in HSI with the use of L-methionine and MHA-Ca supplements. Dietary methionine supplementation is reported to improve amino acid ratio, and it is well known that nutrients balance is crucial to liver size (Hansen et al., 2007). Furthermore, the effective supplementation of methionine in feed has been reported to alleviate fat accumulation in liver (Chiji et al., 1990). In this study, partial fishmeal replacement in the diet NC led to hypertrophied B-cell, which is consistent with results in Burri et al. (2020). Dietary methionine deficiency has been reported to bring about a range of physiological disorders, such as liver damage, and suppression of intestinal epithelial growth (Oz et al., 2007). In previous researches, the histomorphological effects of methionine supplementations on the hepatopancreas have rarely been reported. Normal structure of hepatocytes and regular arrangement of hepatopancreas tubules in the MET and MHA-Ca groups demonstrated the restoration effect of methionine supplementation in low-fishmeal diet on hepatopancreas of *L. vannamei*.

In the present study, dietary supplementations elevated shrimp methionine content to different extents depending on the king of supplements. The divergence in methionine levels *in vivo* appeared to be associated with the slow release and absorption of MHA-Ca, while crystalline methionine has high leaching and rapid absorption rate (Drew et al., 2003; Niu et al., 2018). The addition of crystal methionine and MHA-Ca significantly increased growth performance of white shrimp without significant changes in crude protein content. This phenomenon has also been observed in previous studies (Chen et al., 2018; Wang et al., 2019). The fact that protein deposition was not found in crude nutrients might be related to the organization in which protein synthesis occurred. The utilization of different methionine sources was compared using the relative expression levels of associated key genes. Free methionine is absorbed and used for protein synthesis or is converted to S-adenosylmethionine (SAM), a methyl group donor for the methylation, transsulfuration, and aminopropylation (Anstee and Day, 2012; Machado et al., 2021). In the methionine cycle, the synthesis of SAM requires a key molecule methionine adenosyltransferase (MAT) (Froese et al., 2019). As one of MAT isomerases, *mat2a* is essential to modulate catalytic activity of MAT. In the current study, compared to the higher *mat2a* level in shrimp fed the PC diet, methionine deficiency signals in the NC group caused the reduction of methionine consumption in methylation cycle (Wan et al., 2017). Most marine animals cannot synthesize enough taurine by themselves to fulfil the requirement for healthy growth and development (Tong et al., 2019). In the process of taurine synthesis, the *cdo* is sensitive to flux of nutrient availability and considered as key enzyme that catalyzes cysteine sulfur to derive taurine (Salze and Davis, 2015). In the present study, the *mat2a*, whose expression was unaffected by supplementation of MHA-Ca in diets, and the *cdo* gene, whose expression was elevated, suggested that the absorbed free amino acids might prefer protein synthesis to the methionine cycle. However, the addition of crystalline L-methionine did not promote the expression of *cdo*. Zhou et al. (2021) reported that both DL-methionine and MHA-Ca elevated the expression of *cdo* in common carp (*Cyprinus carpio*). It was generally believed that the differences in gene expression may be caused by methionine levels, metabolic differences, and species.

With the deepening of research, the mTOR signaling pathway is not only considered as a key node in terms of the immune system, but also an amino acid-sensing pathway to regulate cell growth and autophagy (Powell et al., 2012; Tang et al., 2015). When amino acids are sufficient, the pyruvate dehydrogenase system activates the mTOR pathway, further promotes protein synthesis, and suppresses autophagy (Chotechuang et al., 2009; Jewell and Guan, 2013). In contrast, amino acid starvation triggers amino acid response (AAR) pathway and suppresses the nutrient sensing mTOR pathway to arrest cellular proliferation (Tang et al., 2015). In contrast to the results of Zhou et al. (2021), whose work found that the addition of methionine products did not affect the gene expression level of mTOR in common carp (*Cyprinus carpio*), the present study showed that both L-methionine and MHA-Ca supplements led to higher levels of *tor* in the mTOR signaling pathway, albeit still lower than the level of shrimp fed the PC diet. *4ebp1* is a significant translational regulator of mTOR signaling pathway (Fingar and Blenis, 2004). The activation of *tor* by methionine directly phosphorylates *4ebp1*, which enhances protein synthesis and promotes cell growth and proliferation (Hsieh et al., 2010). When amino acid supply is sufficient, Rag family GTPases will facilitate the localization of mTORC1 to the lysosome, thus inducing an increase in mTORC1 activity (Zhu and Thompson, 2019). Although our results indicated no significant changes of *rag c* even with methionine supplementation, it might be able to activate the expression of other Rag proteins. The stimulatory effect of supplementary methionine on mTOR signaling pathway has been demonstrated in previous studies (Su et al., 2018; Farinha et al., 2021; Lu et al., 2021).

In evaluating the difference of methionine products, it is important to ascertain the impact on nutrients parameters and health-related indices to have a more comprehensive assessment. When about 50% fishmeal was replaced, the T-CHO in plasma remained constant in the current study, and methionine supplements did not alter the T-CHO levels. Lu et al. (2021) suggested that the T-CHO levels appeared to be more closely related to fishmeal levels than methionine in diets. The reduction of T-CHO content in plasma in response to methionine deficiency was observed in *L. vannamei* when 65% fishmeal was replaced (Ji et al., 2021), while the T-CHO levels of *L. vannamei* exhibited no alterations when 60% fishmeal was replaced (Wang et al., 2019). As key transaminases in amino acid metabolism, AST and ALT are employed as sensitive hepatic damage biomarkers (Ma et al., 2018; Hu et al., 2021). In this work, higher plasma AST level indicated hepatopancreatic injury in response to low fishmeal. Dietary L-methionine and MHA-Ca supplementations slightly decreased the AST levels. The results of this work suggested the adverse effect of methionine deficiency on the health status of *L. vannamei* by decreasing anti-oxidants including CAT and GSH, as well as by increasing MDA content, which is an indicator of cell damage as the product of lipid peroxidation (Dawood et al., 2017). Our results corroborated the enhancement of antioxidant system due to methionine supplements. Specifically, the addition of 3 g/kg L-methionine boosted the T-AOC level and the gene expression levels of *gpx* and *sod*, while dietary 3 g/kg MHA-Ca supplementation upped GSH of *L. vannamei*. Meanwhile, both additions reduced MDA levels. T-AOC represents the total antioxidant capacity of animal (Hu et al., 2019). CAT, SOD, GSH and GPx are major anti-oxidants that participate in scavenging reactive oxygen species (ROS) (Chen et al., 2020). Methionine takes the forms of cysteine precursor during transsulfuration pathway in GSH synthesis (Machado et al., 2018). It has been reported that methionine supplementation in feed can reduce ROS-induced tissue damage by increasing GSH activity (Del Vesco et al., 2014). Furthermore, methionine supplementation in diets also exerts a positive effect on other anti-oxidants, such as T-AOC, CAT and SOD (Kuang et al., 2012; Zhou et al., 2018; Ji et al., 2021; Song et al., 2021). In this work, it was found that supplementing with L-methionine markedly increased LZM activity of *L. vannamei*. By attacking peptidoglycan in the bacterial wall, lysozyme works as part of the innate immunity of invertebrates against bacterial and viral infections (Paulsen et al., 2001; Li et al., 2015).

Methionine supplementation has been proven to elevate the LZM activity of aquatic animals, such as Jian carp (*cyprinus carpio* var. Jian) and *L. vannamei* (Tang et al., 2009; Kuang et al., 2012; Lu et al., 2021). The onset of apoptosis is commonly associated with caspase expression (Bratton et al., 2000; Reed, 2000). Caspase 3 causes a protease cascade reaction that ultimately leads to apoptosis (Hastak et al., 2003; Sun et al., 2004). Non-differential *casp 3* in *L. vannamei* fed four diets indicated that no severe apoptosis occurred due to fishmeal replacement with plant protein.

## Conclusion

Both 3 g/kg L-methionine and 3 g/kg MHA-Ca supplemented in diets improved the growth performance and methionine content in *L. vannamei*, and ameliorated morphology of hepatopancreas blighted by the low-fishmeal inclusion. Moreover, the growth performance of shrimp fed L-methionine and MHA-Ca supplements was almost no different from that of shrimp fed the high-fishmeal diet. As a result of dietary L-methionine and MHA-Ca supplementations, different anti-oxidants were enhanced in *L. vannamei*. Protein synthesis was facilitated by activating the mTOR signaling pathway in response to the addition of L-methionine and MHA-Ca in low-fishmeal diets, although the expression levels of the relevant genes were still lower compared to those of high-fishmeal diet. Moreover, dietary supplementation with MHA-Ca had a beneficial effect on the taurine synthesis pathway.

## Data Availability

The original contributions presented in the study are included in the article/Supplementary Material, further inquiries can be directed to the corresponding author.
