# Supplementary material for: Comparative study on the effects of crystalline L-methionine and methionine hydroxy analogue calcium supplementations in the diet of juvenile Pacific white shrimp (Litopenaeus vannamei)
Source: Front Physiol. 2023 Jan 30;14:1067354. doi: 10.3389/fphys.2023.1067354 (PMC9923173; doi:10.3389/fphys.2023.1067354)
Supplement: Supplementary file 1 [file Table1.DOCX]

TABLE S1 | The proximate composition of ingredients (% dry matter)

| Ingredients | Crude protein | Crude lipid |
| --- | --- | --- |
| Fishmeal | 66.87 | 8.90 |
| Soybean meal | 43.81 | 2.61 |
| Fermented soybean meal | 48.10 | 1.76 |
| Soybean protein concentrate | 64.88 | 0.48 |
| Shrimp meal | 59.37 | 6.77 |
| Squid liver meal | 44.08 | 15.31 |
| Chicken meal | 67.80 | 12.08 |
| Wheat flour | 10.88 | 1.70 |
